# Nuclear Estrogen Receptors in Prostate Cancer: From Genes to Function

**DOI:** 10.3390/cancers15184653

**Published:** 2023-09-20

**Authors:** Silvia Belluti, Carol Imbriano, Livio Casarini

**Affiliations:** 1 Department of Life Sciences, University of Modena and Reggio Emilia, 41125 Modena, Italy; silvia.belluti@unimore.it (S.B.); carol.imbriano@unimore.it (C.I.); 2 Unit of Endocrinology, Department of Biomedical, Metabolic and Neural Sciences, University of Modena and Reggio Emilia, Ospedale di Baggiovara, 41126 Modena, Italy

**Keywords:** prostate cancer, estrogens, estrogen receptors, alternative splicing, cancer transcript variants, gene regulation, estrogen-receptor-targeting drugs

## Abstract

**Simple Summary:**

Estrogens are steroid hormones that interact with nuclear receptors (ERα and ERβ) and a membrane G-protein-coupled receptor (GPER) to regulate multiple physiological processes. Abnormal ERs and GPER signaling can lead to different disorders, including cancer, making them attractive drug targets. Estrogen-related pathways are implicated not only in breast cancer but also in prostate cancer, providing potential treatment opportunities. New compounds targeting ERs have led to therapeutic advancements, but cancer drug resistance remains a challenge. Genetic and biological mechanisms regulating the expression and activity of nuclear estrogen receptors in prostate cancer are discussed in this review. A comprehensive characterization of specific splice variants in prostate cancer subtypes might lead to new stratification and therapeutic opportunities.

**Abstract:**

Estrogens are almost ubiquitous steroid hormones that are essential for development, metabolism, and reproduction. They exert both genomic and non-genomic action through two nuclear receptors (ERα and ERβ), which are transcription factors with disregulated functions and/or expression in pathological processes. In the 1990s, the discovery of an additional membrane estrogen G-protein-coupled receptor augmented the complexity of this picture. Increasing evidence elucidating the specific molecular mechanisms of action and opposing effects of ERα and Erβ was reported in the context of prostate cancer treatment, where these issues are increasingly investigated. Although new approaches improved the efficacy of clinical therapies thanks to the development of new molecules targeting specifically estrogen receptors and used in combination with immunotherapy, more efforts are needed to overcome the main drawbacks, and resistance events will be a challenge in the coming years. This review summarizes the state-of-the-art on ERα and ERβ mechanisms of action in prostate cancer and promising future therapies.

## 1. Introduction

Estrogens are steroid hormones primarily produced by the ovaries and placenta in females and by the adrenal cortex and testis in males. They play important modulatory and almost ubiquitary roles in physiological and pathophysiological processes by interacting with their two nuclear receptors (ERα and ERβ) and a membrane G-protein-coupled receptor (GPER, also referred to as GPR30) [1]. Nuclear estrogen receptors (ERs) are ligand-dependent transcription factors that regulate gene transcription through estrogen response elements (*EREs*), facilitating normal biological functions of the ligands, while GPER modulates mainly estrogen non-genomic effects. Since abnormal ERs and GPER signaling results in multiple disorders, including cancer, these molecules are interesting targets for pharmacological therapies.

In this review, genetic aspects regulating the role of nuclear estrogen receptors in prostate cancer are discussed.

## 2. Physiology of Estrogens and Their Roles

### 2.1. Estrogen Biosynthesis and Physiological Functions

Estrogens are molecules known since several decades ago [2]. They are synthesized upon aromatization of androgens, and catalyzed by the cytochrome P450 aromatase (P450Aro) that is encoded by the *CYP19A1* gene [3,4]. The aromatase coding gene is located at position 15q21.1 and spans about 75 kilobases. It has multiple transcriptional start sites with individual promoters, leading to an extremely tissue-specific regulation of the transcription modulated by several hormones and other molecules [5,6,7]. Relatively high expression levels of aromatase transcripts are found in ovarian granulosa cells from women of a fertile age and, to a lesser extent, in the testis of men [8]. Moreover, human extragonadal sites of estrogen production were described, such as the placenta, adrenocortical cells, adipocytes and stromal cells, osteoblasts, fibroblasts and keratinocytes, smooth muscle cells, hippocampus, and hypothalamus [8]. Aromatase expression is assumed to be a rate-limiting step for estrogen synthesis, although weak androgen aromatization depending on the steroidogenic acute regulatory protein (StAR) enzyme was detected in adolescent females [9].

Estrogens are commonly known to induce the feminization of girls during adolescence, occurring under the stimuli of gonadotropins and in the absence of effective opposing action from androgens. Moreover, during the fertile age of women, estrogens are known to regulate the ovarian follicle development and growth, acting as proliferative and anti-apoptotic factors supporting oocyte maturation [10,11]. These hormones are synthesized in ovarian granulosa cells in response to follicle-stimulating hormone (FSH) stimulation, which triggers aromatase activity [12,13], and supported by luteinizing hormone (LH)-induced androgen production [14,15]. Effective estrogen production is ensured with the collaborative interaction between ovarian granulosa cells, which express FSH receptors (FSHR) and surround the oocyte, together with theca cells, which have androgenic capability and LH/choriogonadotropin (hCG) receptors (LHCGR) [16]. Finally, the placenta produces estrogens during pregnancy, primarily estriol and estradiol, by aromatizing fetal steroids of an adrenal origin [17,18]. A deficit of estrogens may lead to a range of symptoms [19,20] at different grades that, at the level of the ovary, may be identified as an irregular menstrual cycle or amenorrhea [21,22,23]. Also, a deficit of estrogens is classically associated with an impaired bone metabolism and increased risk of osteoporosis [24], since these hormones regulate bone cell functions and the remodeling of this tissue [25,26]. Interestingly, these effects occur even in men, since estrogen replacement therapy restores normal biochemical measures of bone metabolism in men affected by aromatase deficiency [27].

The adrenal gland is another site for estrogen production in both sexes [28]. Here, these steroids could have a role in the regulation of the renin–angiotensin–aldosterone system, contributing to the physiological maintenance of blood pressure and hydromineral balance [29,30]. Moreover, estrogens may support the development of the adrenal cortex during fetal life, thanks to the interplay between this structure and the placenta [31]. The adrenal contribution to circulating levels of these hormones has been debated for a long time [28], given that estrogens synthesized within extragonadal compartments do not achieve relatively high concentrations and are likely only active locally through paracrine or intracrine action.

Other sites of estrogen synthesis could produce a minor contribution to the overall serum levels of these hormones. Among all, the estrogenic potential of adipose tissue cells and of the brain is well known [32,33,34], since they have aromatase expression [35]. On one side, the adipose tissue works as the main storage site for these sex steroids [36,37]. On the other side, estrogens impact adipose tissue distribution via paracrine action [38], modulate adipocyte functions and metabolism [39], and are responsible for an increased breast cancer risk [40,41,42,43,44,45].

In the brain, estrogens are involved in the regulation of cognitive functions and stimulate spinogenesis and synaptogenesis in regions such as the prefrontal cortex, hippocampus, basal forebrain, thalamus, cerebellum, and brainstem [46,47]. These actions vary according to age and, in women, to the menstrual cycle stage [46]. The role of estrogen in the central nervous system is not limited to sexual dimorphic differences. Rather, these molecules are involved in the processing of sensory information, regulation of affective behavior, synaptic plasticity, learning and memory [48], and neuroprotection against inflammatory damages [49]. During the fetal life, these molecules play a key role in determining sexual differentiation of the brain and subsequent sex differences in behavior, according to the aromatase expression pattern [50,51]. Taken together, these data reveal the central role played by estrogens in regulating brain functions, outlining the need to explore their potential as molecules with neuroprotective properties [52] and in the prevention of brain pathologies such as Alzheimer’s disease [53]. However, the clinical relevance of estrogen action in the brain is still largely unclear and needs further research.

### 2.2. Role of Estrogens in the Prostate

In men, germ cells, sperm, and Leydig and Sertoli cells display aromatase expression, and the testis is indeed one of the main estrogen-producing glands in males [54]. These cells serve as the major source of estrogens in the male reproductive tract. Therefore, estrogens are also found in the semen [55]. However, evidence demonstrating that estrogens have a major direct role in adult testicular functions is poor [56]. Pioneering data were provided by the study of a very rare case of male estrogen deficiency, which described an adult man with defects in bone and glucose metabolism, but normal pubertal development, masculinization, and sexual function [57,58]. Serum estrogen levels were elevated while testosterone concentration was normal, as an indication of an ER-coding gene disruptive mutation confirmed with a genetic analysis. Other informative results were obtained from men affected by an aromatase deficit caused by *CYP19A1* gene loss-of-function mutations who, again, had normal pubertal development and masculinization in addition to metabolic and bone clinical issues [27,59]. Therefore, the role of estrogens in spermatogenesis and testicular functions is still largely unknown, although parameters of sperm quality in infertile men might be sensitive to administration of selective estrogen receptor modulators (SERMs) [60] or estrogenic disruptors [61]. A synergistic effect with androgens, likely exerted through membrane receptors, has been proposed [62], but this matter is still unclear.

Over the years, high relevance has been given to the role of estrogens in males [63]. For instance, these hormones have a role in modulating tissue homeostasis and cell proliferation in the prostate, where they can stimulate as well as inhibit growth, depending on the predominant ERα- or ERβ-mediated effects [64,65]. In general, it is assumed that excessive ERα activation is linked to aberrant proliferation, inflammation, and development of premalignant lesions, while these signals are counterbalanced by anti-proliferative effects exerted through ERβ [64]. Therefore, these steroids contribute to the maintenance of prostate functions, such as the production of the seminal liquid. Several studies agree with the view that estrogens exert their action through both direct receptor-mediated action and indirect effects, by altering the whole endocrine status [66]. Informative results were provided using genetically modified mouse models, such as aromatase-knock-out (KO) or over-expressing mice (ArKO and AROM+), and the ER-KO (αERKO and βERKO). Only prostatic enlargement occurring with age was found in αERKO mice, while βERKO and ArKO displayed prostate hyperplasia. AROM+ even developed squamous metaplasia and had elevated serum estradiol levels, although these results were not univocally confirmed [66]. In men, aromatase is physiologically expressed in the stroma of the prostate, which is capable of local estrogen biosynthesis [67]. However, aberrant expression of the aromatase protein was found in other prostate cells, confirmed in prostate cancer (PCa) cell lines and even in cases of benign prostatic hyperplasia [7,68]. In these tissues, disregulation of aromatase expression would be due to alteration of *CYP19A1* promoter usage [67]. Given the clear estrogenic nature of certain prostate tumors, the use of aromatase inhibitors and SERMs as an endocrine therapy for prostate cancer has been proposed since a long time ago [69,70]. The role of nuclear estrogen receptors in PCa is discussed in the next sections.

## 3. Nuclear Estrogen Receptors

Estrogens regulate several physiological functions mediated by their two nuclear receptors. In humans, the *ESR1* gene is located at locus 6q25.1 on chromosome 6 and encodes for the full-length ERα protein, consisting of 595 amino acids (66/67 kDa) [71,72]. The ERβ protein of 530 amino acids (59 kDa) is encoded by the *ESR2* gene, which is located on chromosome 14 at locus 14q23.2 [73,74]. These molecules have similar binding affinity for the two main ligands, estradiol and estrone, and act as ligand-activated transcription factors for target genes in the cell nucleus.

As other nuclear receptors, ERs have a modular structure where multiple functional domains have specific functions, namely an N-terminal transactivation domain (NTD/AF1), a DNA-binding domain (DBD), a hinge region, a C-terminal cofactor interaction domain, and a ligand-binding domain (LBD). The NTD/AF1 contains zinc-finger domains that allow the binding and transactivation of target genes. The NTD is one of the most significant structural differences between ERα and ERβ, having approximately 15% sequence homology [75]. The DBD allows ERs to bind estrogen response elements (EREs) in target genes, while the hinge region contains nuclear localization signals and can bind to molecular chaperones. The LBD/AF2 carboxyl terminus encompasses binding sites for co-activators, co-repressors, and the estrogen-binding region, together with a ligand-dependent activation domain (AF2). In particular, natural ligands bind a cavity-shaped domain of the receptor, which may be targeted by several other molecules with agonistic and antagonistic behaviors [76].

New insights into the molecular structure of ERα were provided using the resolution of its structure in a complex with DNA and co-activators using cryo-electron microscopy (EM) [77]. These data provided large advancements compared to what was carried out with the crystallographic structure resolved previously [78]. Cryo-EM depicted the molecular mechanism of action with which ERα binds the *ERE* DNA as a dimer. Then, the steroid receptor co-activator 3 (SRC-3) protein is independently recruited, via the transactivation domain, by each of the two ligand-bound ERα monomers and together with activation function 1 (AF-1). In turn, the two SRC-3s interact with different regions of one secondary co-activator (p300) protein through multiple contacts [77]. These data are of high relevance to elucidate the mechanisms with which active nuclear receptors upregulate target gene transcription.

ERs carry highly polymorphic regions that may impact the activity of estrogens. This is not surprising, since ER-coding genes are evolutionarily old and unstable, predisposing these sequences to repeated mutations over time [79], and leading to aspecificity for their ligands [80] and capability to bind even plant-derived molecules, such as flavonoids [81]. For instance, the *ESR1* gene carries single-nucleotide polymorphisms (SNPs) linked to estrogen resistance [82], breast cancer [83,84], mineral bone density [85], and cardiovascular risk factors [86]. CA nucleotide repeats were found in the genomic region containing the *ESR2* gene, suggesting it could be used as a marker of endocrine functions [87] and could be associated with blood pressure [88], Alzheimer’s disease [89], risk of hypospadias [90], and osteoarthritis [91]. Five more receptor variants were identified upon screening of *ESR2* 5′ and 3′ untranslated regions, although no association with specific phenotypes or pathologies was found [92]. Other *ESR2* SNPs that are linked with increased transcriptional activation were related to disorders of sexual and pubertal development [93,94]. In summary, ER-coding genes are highly unstable, leading to high susceptibility to mutations and subsequent modulation or impairment of receptor functionality.

**Figure 1:**
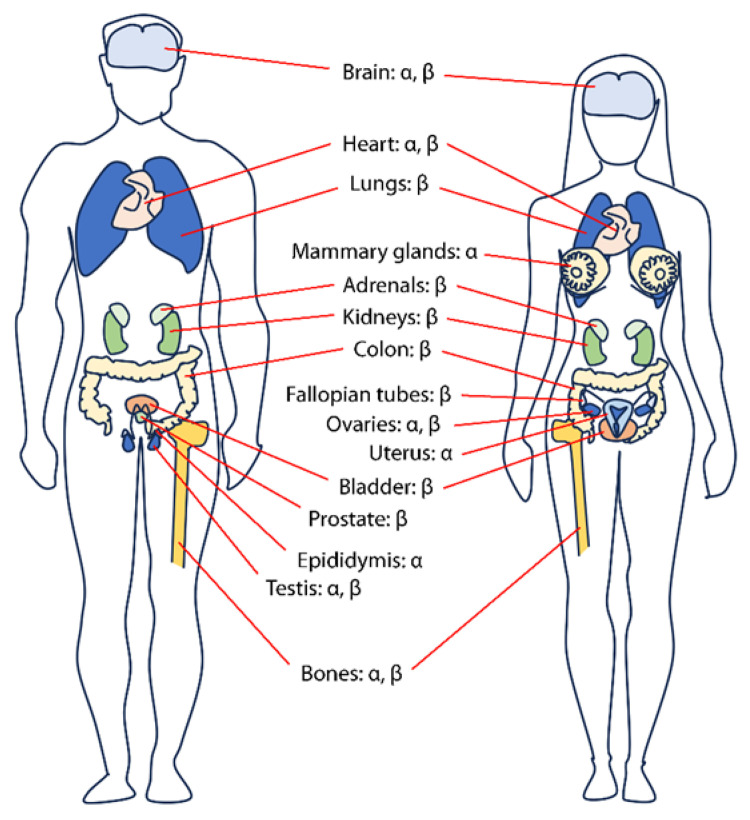
Representation of ERα and ERβ sex-specific tissue distribution. The picture is not indicative of absolute receptor expression levels.

In the body, tissue-specific expression patterns (Figure 1) and the ratio between the two receptors determine the sensitivity of the target tissue to estrogens and their local effects [95]. Although these receptors are expressed almost ubiquitously, ERα is preferentially expressed in the cardiovascular and adipose tissues, thymus, mammary gland, and uterus. In contrast, ERβ is prevalently expressed in the ovaries, prostate, testis, and bone, while similar expression levels could be detected in the brain, hypothalamus, pituitary, and liver [95,96,97]. The tissue distribution of ERs is one of the determinants to understand their differential response to agonists triggered upon interaction with the ligand-binding cavity [76]. In fact, it is well known that ERα and ERβ mediate different, or even opposite, estrogen-induced conformational changes [98] that lead to distinct effects at the cellular level, depending on the site of action and receptor expression levels and sub-type [99].

## 4. Expression and Splice Isoforms of Estrogen Receptors in Prostate Cancer

### 4.1. Estrogen Receptor Expression in Prostate Cancer

Prostate tissues express both ERα and ERβ estrogen receptors. ERβ is predominantly expressed in prostate epithelial cells, particularly luminal cells [100], whereas ERα is mostly expressed in stromal cells and basal prostate epithelial cells [74,101].

Until now, it has been hypothesized that ERβ has a primarily protective effect in PCa, whereas ERα could be potentially oncogenic. However, the function of ERs in PCa is still debatable, as contradictory findings regarding their behavior in PCa progression are still emerging.

Using publicly available genomic datasets, the prevalence of *ESR1* and *ESR2* gene mutations or copy number variations in patients with metastatic or advanced PCa was 4% (5/150) compared to 2% (11/492) in patients with early PCa [102]. Interestingly, the prevalence of ER aberrations in patients with neuroendocrine PCa (NEPC) was considerably higher than in patients with early PCa (17% vs. 2%; *p* < 0.05). The most prevalent abnormality was an increased copy number. In patients with early PCa, an altered ER copy number or mutation was associated with shorter overall survival (*p* = 0.01), but there was no significant difference in progression-free survival [102]. In addition, a large population-based case–control investigation revealed an association between an SNP in the promoter region of the *ESR2* gene and the risk of developing PCa [103].

In high-grade prostatic intraepithelial neoplasia (hg-PIN), ERα protein and mRNA levels are upregulated, and its expression spreads from basal to luminal cells. Immunohistochemistry and mRNA in situ hybridization experiments indicate that ERα is upregulated in high-grade Gleason score (GS) tumors compared with those with low GS, and in recurrent carcinomas, likely mediating the carcinogenic effects of estradiol. Nevertheless, contrasting results were obtained about ERα protein and mRNA expression in castration-resistant prostate cancers (CRPC) [101,104,105,106]. In addition, inconsistent findings were obtained concerning the transcriptional regulation of ERα. Indeed, the *ESR1* gene promoter was found to be extensively methylated in PCa cell lines and tissues, with a positive correlation between methylation levels and tumor pathological grade [107,108]. In contrast, another study found no direct correlation between promoter methylation and mRNA levels of *ESR1* in PCa data from The Cancer Genome Atlas (TCGA) [109]. High expression levels of ERα protein and mRNA were associated with shorter progression-free interval and shorter biochemical recurrence-free survival, respectively. Yet, the presence of ERα protein, as determined with IHC, was also associated with the efficacy of endocrine therapy [109,110,111,112,113,114,115] and high ERα expression has been identified in tumor stroma cells, and this correlates with a better prognosis for PCa patients [116,117,118]. These contradictory results make it difficult to draw conclusions regarding ERα expression and relevance in advanced tumors.

In contrast, ERβ is partially lost in hg-PIN and downregulated in approximately 50% of localized and CRPC tumors. Decreased ERβ expression may affect both stromal and epithelial cancer cell development, and PCa tissue staining revealed that ERβ expression is inversely correlated with the progression of PCa to a high Gleason’s grade. High levels of CpG methylation in the proximal promoter region of the *ESR2* gene contribute to its downregulation in PCa [119]. It is widely accepted that ERβ may inhibit the development of PCa by acting as a tumor suppressor. However, many findings have questioned this latter concept [120]. To depict a clear picture of the role of ERs in the progression of PCa, it is necessary to consider the experimental pitfalls caused by the available ER antibodies and the significance of various ER splicing variants [121,122,123].

### 4.2. ER Isoforms in Prostate Cancer

**Figure 2:**
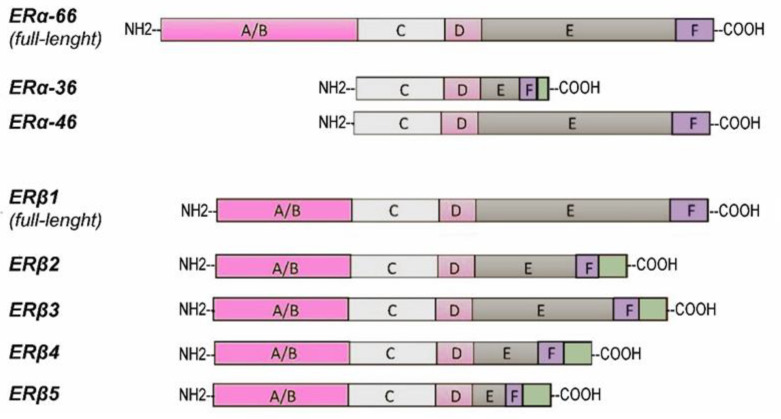
Schematic structures of estrogen receptors’ (ERs) isoforms. Different functional domains are highlighted: the N-terminal transactivation domain NTD/AF-1 (A/B), the DNA-binding domain (C), a hinge region (D), and the C-terminal cofactor interaction domain AF-2 and a ligand-binding domain (LBD) (E/F).

It is well known that specific splice variants of transcription factors modulate the balance between cellular processes that enhance or inhibit tumorigenesis and cancer progression [124]. This was also observed in PCa [125,126,127]. Human ER variants were identified in testis and prostate cells (Figure 2), as well as in breast and other cancer cell lines. In addition to the full-length ERα-66, several isoforms of ERα resulting from alternative gene splicing have been identified, including ERα-46 and ERα-36. A 46-kDa ERα isoform was cloned and described as a receptor splicing variant lacking 173 N-terminal amino acids, including AF-1 [128,129]. It resulted in a ligand-independent transactivation domain, which retains both hormone- and DNA-binding capability, and may be targeted at the cell membrane and work as a dominant-negative inhibitor of ERα activity.

The ERα-36 transcript is initiated by an alternative promoter within the first intron of the *ESR1* gene [130,131,132]. ERα-36 differs from ERα-66 in that it lacks both transcriptional activation domains (AF-1 and AF-2) while retaining the DNA-binding domain, partial dimerization domain, and ligand-binding domain. It features a unique 27-amino acid domain that replaces the final 138 amino acids encoded by exons 7 and 8 of the ERα-66 gene. ERα-36 is primarily expressed on the plasma membrane and in the cytoplasm, where it mediates membrane-initiated estrogen signaling effects, such as activation of the mitogen-activated protein kinase/extracellular-signal-regulated kinase (MAPK/ERK) signaling pathway, and stimulates cell growth. ERα-36 has been found in the prostate tissue of both healthy and PCa-affected individuals [102].

Regarding ERβ, five splicing isoforms (ERβ1, -2, -3, -4, and -5) were identified three decades ago [133,134]. Human ERβ splice variants originate from the alternative splicing of exon 8, resulting in sequence variations or protein truncations in the C-terminal regions. The full-length ERβ1 (often referred to as ERβ) and the truncated ERβ2 and ERβ3 have a length ranging between 495 and 530 amino acids and mainly differ from each other in helix 10 of the ligand-binding domain (LBD). ERβ4 and ERβ5 are characterized by a portion of the C-terminal LBD upstream of unique sequences.

These variations lead to the shortening of the LBD and the loss of the AF2 function. Consequently, ERβ1 is the only isoform with ligand-binding ability, whereas truncated ERβ cannot bind estrogens. However, ERβ1 may form heterodimers with ERβ4 or ERβ5 and these molecular complexes have greater transcriptional transactivation activity than the ERβ1 homodimer [135,136]. All these variants are expressed almost ubiquitously, except for ERβ3, which is relatively rare and is mainly restricted to the testis [134], although all of them have a tissue-specific preferential pattern. ERβ2, ERβ3, ERβ4, and ERβ5 have been detected in prostate and PCa samples. While ERβ2 is expressed in both basal and luminal epithelial cells, ERβ5 is almost exclusively found in the basal compartment.

Drug resistance and apoptosis in PCa have been linked to ERβ splice variants [102]. In contrast to ERβ1, which has tumor growth-suppressive effects, ERβ2 and ERβ5 promote tumor growth, inducing stem cell properties and the development of chemoresistance in PCa, whereas ERβ1 might improve cancer cell responsiveness to chemotherapeutic agents [137]. Generally, multiple splice variants are co-expressed in PCa cells; hence, the expression ratio between splice variants is an important indicator of clinical treatment success.

## 5. Functions of Estrogen Receptors in Prostate Cancer Cells

Estrogen-regulated gene products physiologically control proper autophagy, proliferation, apoptosis, survival, differentiation, and vasodilation. Although the androgen receptor (AR) is a leading player in PCa pathogenesis and exerts its functional effect mainly through transcriptional pathways (recently reviewed in [138,139,140]), ERs have been linked to PCa occurrence, development, and prognoses in several studies (Table 1) and PCa risk is correlated with serum estrogen levels. This suggests that estrogen and ERs may be risk factors. Previous studies showed that ERα is upregulated during malignant transformation of the prostatic epithelium, high-grade and metastatic PCa, and CRPC, where androgen-deprivation therapy increases ERα expression, suggesting its oncogenic role [101]. ERα, expressed in prostate stromal tissues, increases prostatic epithelial proliferation via growth factors like basic fibroblast growth factor (bFGF), epidermal growth factor (EGF), and insulin-like growth factor 1 (IGF-1) [141]. In experimental carcinogenesis with testosterone and estradiol, ERα-knockout mice (KO) did not develop hg-PIN or PCa [142], suggesting that ERα promotes prostate epithelial tumors.

Unlike ERα, ERβ is mostly expressed in epithelial cells; therefore, estrogens directly affect the prostate epithelium and protect it against malignancy via ERβ activity [101]. Hyperplastic alterations decrease ERβ expression in luminal prostatic epithelial cells. ERβ is decreased in high-grade PIN and lost in high-grade PCa and after androgen-deprivation therapy (ADT), suggesting that it interferes with tumors. Grade 4/5 carcinomas have lower ERβ levels than grade 3 carcinomas [100,148]. Exogenous ERβ overexpression in PCa cells showed anti-proliferative, anti-invasive, and pro-apoptotic effects [149]. PCa xenograft studies showed that 17beta-estradiol supplements given to ovariectomized female mice restrict tumor establishment and growth with androgen-independent mechanisms [150], suggesting that estrogen therapy could be beneficial for CRPC. Moreover, hg-PIN occurs in ERβ-knockout (βERKO) mice due to increased proliferation, reduced apoptosis, and accumulation of poorly differentiated cells [151,152]. The above findings suggest that ERβ1 is crucial for prostate health and tumor suppression. Although it is diminished in PCa above Gleason grade 3, ERβ is a possible therapeutic target in the early stages of the disease. As further evidence, ERα activation in βERKO mice triggers abnormal proliferation, inflammation, and premalignant lesions, while ERβ activation in αERKO mice is essential for prostatic stromal–epithelial cell signaling and mediates anti-proliferative effects that counteract the proliferative effects of androgens on the epithelium.

Intensive research on the expression of ER and its variants in the human prostate has revealed that ERβ1 is lost during cancer progression, although its splice variant ERβ2 is expressed in advanced PCa [144]. In PCa PC3 cells stably expressing ERβ1 or ERβ2, ERβ1 decreased proliferation and bone-metastasis-associated factors, whereas ERβ2 boosted proliferation and upregulated them [145]. Consequently, in PCa cells, ERβ2 possesses oncogenic properties that are in opposition to ERβ1 tumor-suppressing effects.

### 5.1. Transcriptional Activity of ERs

Many, but not all, estrogen-responsive genes include an *ERE* sequence in their regulatory regions that can be directly bound by ligand-associated ERs as homodimers or heterodimers [153,154,155]. To mediate transcription regulation, ERs undergo conformational changes upon ligand binding and then interact with numerous co-activators and co-repressors of transcription, such as members of the general transcription factor apparatus and chromatin-remodeling proteins [156]. The transcriptional responses can be fine-tuned to meet specific physiological requirements by recruiting certain groups of co-activators or co-repressors. In addition, ER proteins can indirectly regulate genes without *ERE*-like sequences by interacting with other transcription factors. Activator protein-1 (AP-1), nuclear factor-κB (NF-κB), stimulating protein-1 (Sp-1), activating transcription factor (ATF)-2/c-jun, ATF-2/cAMP response element-binding protein (CREB), ATF-1/CREB, and nuclear transcription factor Y (NF-Y) are examples of intermediate factors that can mediate ER recruitment to promoters or enhancers. About 35% of the annotated human estrogen-responsive genes are regulated with indirect ER-DNA interactions [157].

According to transcriptional studies [158,159], estrogen stimulation both activates and inhibits the transcription of ER target genes. Positive regulators of cell proliferation, such as growth factors and cell cycle regulators, are often upregulated genes. Many of the downregulated genes work in the opposite direction, inhibiting the cell cycle or inducing apoptosis [158]. This gene expression signature is consistent with the concept that estrogen stimulates cell survival. Moreover, recent analyses pointed out that estrogen receptor signaling could be associated with neuroendocrine-like tumors [160].

Estrogen signaling via ERα increases with PCa progression and can induce crucial oncogenic events. While the effects of ERα signaling on breast cancer have been thoroughly studied (recently reviewed in [161,162,163]), the function of this nuclear receptor in prostate pathophysiology is less well understood. Evidence for the presence of a functional ERα-signaling network in PCa includes ERα-mediated regulation of the oncogenic transmembrane protease, serine 2 (TMPRSS2)-Ets-related gene (ERG) fusion protein. ERα is preferentially recruited to intergenic regions of the prostate genome, as determined with a ChIP-seq analysis of global ERα binding in PCa cells [106]. Transcriptome sequencing data and binding profile comparisons revealed that ERα may control the expression of non-coding RNAs. Expression of the long noncoding RNA (lncRNA) nuclear paraspeckle assembly transcript 1 (NEAT1) is regulated with functional ERα signaling, and cancers of the prostate that originate in NEAT1-positive epithelial cells are resistant to androgen inhibitors and androgen deprivation.

Differently from ERα, several studies characterized the role of ERβ as a major tumor regulator in PCa, with a role in controlling cell proliferation and metabolism through both direct and indirect mechanisms. Dey and colleagues [164] found that ERβ regulates apoptosis in PCa cells by upregulating the transcription of forkhead box O3 (FOXO3a), which in turn increases the p53-upregulated modulator of apoptosis (PUMA) in a p53-independent manner and triggers apoptosis via the intrinsic pathway and caspase-9. In advanced CRPC cell lines, the upregulation of ERβ levels can significantly suppress transforming growth factor β 1 (TGF-β1) and IGF-1 expression which, in turn, reduces the expression of downstream anti-apoptotic proteins B-cell lymphoma 2 (Bcl-2) and Survivin, and induces apoptosis [165].

Epithelial hyperplasia and enhanced expression of AR-regulated genes are among the traits observed in the prostates of mice with ERβ inactivation. These genes are also increased in PCa. AR and ERβ are crucial co-regulatory receptor proteins in PCa, and their interactions and crosstalk in signaling have a major influence on PCa pathogenesis. Repression of AR activity by ERβ is associated with tumor suppression in PCa [166]. Therefore, it is essential for PCa therapy to preserve the dynamic equilibrium between AR and ER. ERβ downregulates AR signaling, inducible nitric oxide synthase (NOS), antioxidant glutathione peroxidase 3 (GPX3), and interleukin (IL)-6, thus reducing inflammation and cell proliferation. It has been established that ERβ regulates AR signaling by upregulating the AR co-repressors Dachshund family transcription factor 1 and 2 (DACH1/2) and downregulating the nuclear receptor RORc, which recruits co-activators to the AR promoter and activates AR expression [167]. In line with these observations, it has been demonstrated that ERβ agonists suppress AR expression in androgen-dependent metastatic vertebral cancer of the prostate (VCaP) cells, leading to lower cell survival and increased apoptosis [168].

Moreover, ERβ induces anti-tumoral activity in PCa cells by increasing the expression of anti-proliferative genes like phosphatase and tensin homolog (*PTEN*), *FOXO3a*, Kruppel-like factor 5 (*KLF5*), and cyclin-dependent kinase inhibitor 1A (*CDKN1A*, p21) and 1B (*CDKN1B*, p27), as well as decreasing the expression of genes like phosphatidylinositol 3-kinase (*PI3K*), F-box protein p45 (*SKP2*), *c-MYC*, and cyclin E (*CCNE*), or the oncogenic *TMPRSS2-ERG* fusion. In PCa cells, ERβ activation can also suppress the effects of ERα and induce cell apoptosis [101,167,169].

In PCa, maintenance of the epithelial phenotype and repression of mesenchymal traits is an important function of ERβ and its ligand 5alpha-androstane-3beta,17beta-diol (3beta-adiol), a dihydrotestosterone metabolite that does not bind AR, but efficiently binds ERβ [170,171]. Epithelial–mesenchymal transition (EMT) inducing stimuli, such as TGF-β and hypoxia, reduce ERβ expression, and ERβ loss is sufficient to establish an EMT. ERβ1 destabilizes hypoxia-inducible factor (HIF)-1 protein through proteasomal degradation and represses HIF-1α-mediated transcription of vascular endothelial growth factor (VEGF)-A [170]. In addition, under hypoxic circumstances, ERβ1 directly represses VEGF-A transcription via the *ERE*. In contrast, the ERβ variants ERβ2 and ERβ5 can interact with and stabilize the HIF-1α protein and induce hypoxic gene expression under normoxic conditions [172]. Moreover, the expression of metalloproteinase-2 (MMP-2), VEGF, and other important proteins for invasion and migration is increased, together with the reduction in ERβ expression, resulting in higher invasion and migratory capacity of PCa cells. This finding was further confirmed in a mouse model, where the volume of transplanted tumors and their capacity to metastasize were both considerably higher in the ERβ-silenced nude mouse model than in the control group [146]. In the prostate, ERβ expression correlates with E-cadherin levels [173], and ERβ is a negative regulator of inflammation, which are well-recognized factors in carcinogenesis and metastasis. When ERβ is oxidized by reactive oxygen species, DNA binding is lost and production of E-cadherin is decreased, suggesting that ERβ transcriptional activity is vulnerable to oxidation arising from tissue inflammatory processes [174,175].

### 5.2. Non-Transcriptional ER Signaling

Estrogens control a non-transcriptional signaling pathway by binding to membrane-bound ERα and ERβ, which rapidly regulate ion channel opening or the activation of related enzymes including Ca^2+^ mobilization, PI3K, and mitogen-activated protein kinase (MAPK). This process does not rely on gene regulation and occurs within seconds to minutes, resulting in a rapid non-genomic effect.

Multiple estrogen receptors, including ERα, ERα-36, and ERβ, have been implicated in the non-genomic responses to estrogens [176]. Extensive evidence indicates that a small reservoir of ERα exists outside of the nucleus, at or near the plasma membrane of tumor cells. A small proportion of ERα is palmitoylated in the absence of hormone stimulation, triggering ERα signaling at the plasma membrane via its interaction with caveolin-1. ERα is depalmitoylated in response to estrogen stimulation and initiates downstream phosphorylation cascades via direct interactions with multiple proteins, including Src tyrosine kinases and PI3K. Other regulatory proteins, such as the adaptor protein Crk-associated substrate (CAS, p130Cas) and the focal adhesion kinase (FAK), have been identified as components of the non-genomic complex. This complex activates the Src/MAPK and PI3K/AKT serine–threonine kinase (AKT) pathways, which regulate cell proliferation and survival. Moreover, membrane/cytoplasm-localized ERs may activate signaling cascades via Galectin-3 and non-phosphorylated β-catenin in androgen-independent prostate cancer cell lines (PC3, DU145), thus modulating proliferation, migration, invasion, and anchorage-independent growth of these cells [177].

During the cell cycle, ERβ1 and ERβ2 isoforms are differentially regulated in LNCaP cells. Unlike ERβ2, which is predominantly expressed in the G2/M phase, ERβ1 induces a cell cycle arrest in the early G1 phase in response to estradiol via a non-genomic pathway involving c-Jun N-terminal kinases (JNK). Specifically, the interaction of ERβ1 with JNK is associated with a decrease in c-Jun phosphorylation and, as a result, an inhibition of the c-Jun/AP-1 complex activity that controls cyclin D1 expression [178]. In addition, in several studies on CRPC cells, it was observed that silencing of ERβ leads to phosphorylation of extracellular signal-regulated kinases 1 and 2 (ERK1/2), activates the ERK1/2 signaling pathway, and promotes cell proliferation, while decreasing the ratio of G0/G1 phase cells and apoptosis [74,179,180].

**Figure 3:**
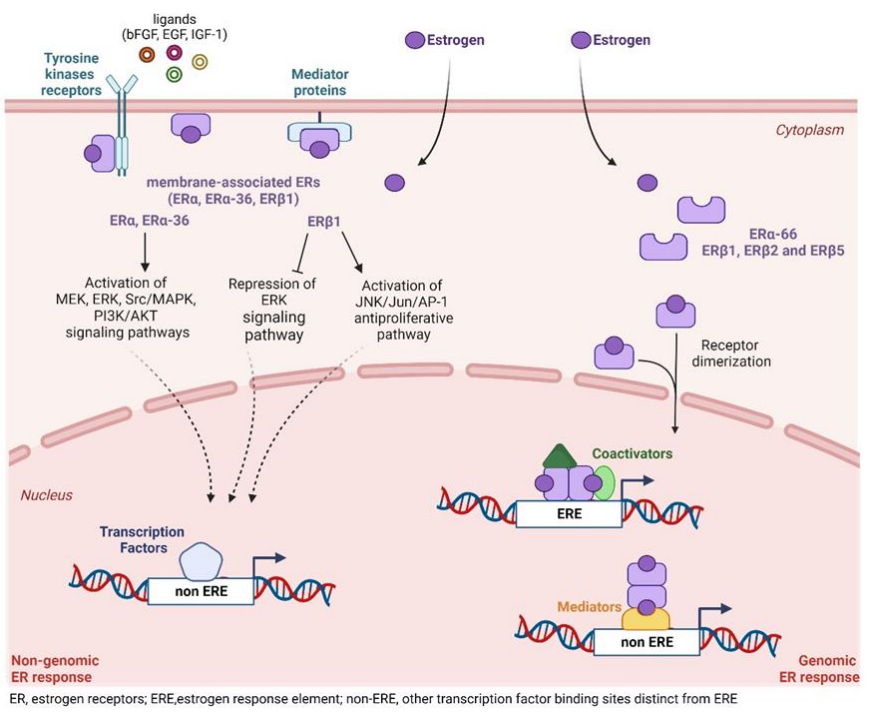
ERs can act through genomic and non-genomic pathways in PCa. In the genomic pathway, estrogen triggers the dimerization of ERs and their nuclear translocation to regulate the transcriptional activity of genes. ERs can directly control the transcription of genes bearing the estrogen response element (*ERE*) in their promoter region. In addition, ERs can modulate gene transcription through protein−protein interactions with other transcription factors (TF), which can mediate the recruitment of ERs at promoters without *ERE*s. In the non-genomic pathway, membrane-associated ERs can participate in signal transduction and modulate the activation of key enzymes and molecular pathways, thus resulting in the indirect regulation of transcriptional programs. Known ER-regulated transcriptional programs in PCa: (I) ERα: transactivation of proliferative, apoptotic, survival, differentiation, vasodilation, autophagy, and inflammation targets; transactivation of oncogenes and non-coding RNAs. (II) ERβ1 transactivation of apoptotic, anti-proliferative, anti-invasive, and anti-inflammatory targets; repression of AR, oncogenes, and hypoxic genes. (III) ERβ2 and ERβ5—stabilization of the HIF-1α protein and induction of hypoxic genes. Created with BioRender.com, accessed on 10 July 2023.

Rapid activation signaling of ERs in the extranuclear region activates multiple signaling pathways, including MAPK/ERK, making endocrine therapy a promising treatment for PCa by blocking pathways associated with extra-nuclear ERβ. ER-mediated transcriptional and non-transcriptional activities in PCa are summarized in Figure 3.

## 6. Molecules Targeting Nuclear Estrogen Receptors

Estrogens and their receptors are recognized targets for therapeutic strategies against ER-positive tumors. Several drugs capable of disrupting the molecular machinery for estrogen synthesis were developed, such as the aromatase inhibitors letrozole, anastrozole, and exemestane [181,182]. ERα-specific drugs, such as selective ER modulators (SERMs), selective ER degraders (SERDs), complete ER antagonists (CERANs), selective ER covalent antagonists (SERCAs), and proteolysis-targeting chimera (PROTAC) ER degraders, are used to counteract the effects of estrogens, especially in breast and uterus cancers, where this receptor triggers mainly proliferative signals [162]. Tamoxifen was the first nonsteroidal anti-estrogen [183]. It belongs to the SERM family and was initially developed for contraception purposes, before becoming a largely used anti-cancer agent for ERα-positive tumors of the breast and uterus. In these tissues, tamoxifen displayed mixed pharmacological behavior, acting differentially as an antagonist and an agonist, depending on ER expression levels [184,185]. In early-stage clinical trials, the anti-estrogen fulvestrant and ERα antagonist toremifene showed potential anti-tumor activity in PCa [186]. Fulvestrant acts as a pure ERα antagonist and belongs to the broad class of SERDs [187], enhancing the degradation of the receptor by blocking the interaction between ERα and the co-regulator AF-2, although other molecular mechanisms of action were described [188,189]. This drug has greater efficacy than tamoxifen and higher binding affinity to ERα than estradiol [190]. Interestingly, fulvestrant and other anti-estrogens demonstrated potential anti-tumor activity in PCa also through ERβ-dependent mechanisms [191]. This effect was exacerbated in those cells positive to ERβ, but not to Erα [192], likely via combined downregulation of *ESR2* and *AR* transcripts [193,194]. These data should be interpreted carefully, since fulvestrant lead to contradicting results in clinical studies of men with castration-resistant PCa [194,195]. Moreover, increasing interest is given to GPER as a target for anti-cancer drugs [196,197], since it may drive cell proliferation via molecular mechanisms depending on nuclear ER co-expression [198,199,200].

**Figure 4:**
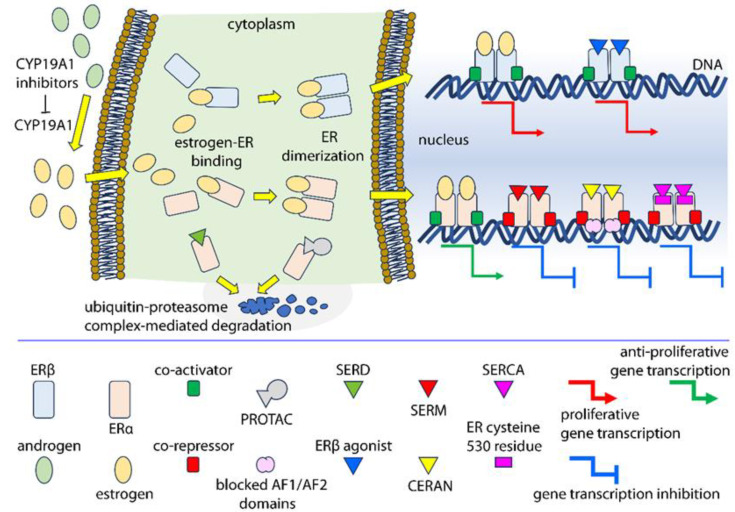
Anti-estrogen molecules’ mode of action. Androgens are the substrate for estrogen production, mediated by the aromatase (CYP19A1) enzyme. Estrogens bind and activate ERs, thus regulating target genes’ transcription. The synthesis of estrogens may be blocked by specific inhibitors. SERDs are ER antagonists that bind the receptor, block its migration to the nucleus, and inhibit receptor-ERE interaction. SERD-ER complexes are degraded by the proteosome. PROTACs are bispecific ligands binding both ER and the E3 ubiquitin ligase, which, in turn, mediates the ubiquitination of the complex, leading to its degradation. SERM competitively binds ERs. The SERM-ER complex can interact with ERE sequences and co-repressors, inhibiting the transcription of target genes. However, this effect is tissue-specific and co-activators may be recruited in certain tissues, such as the endometrium and bone. CERANs exert antagonistic action by blocking AF1 and AF2 transcriptional activation domains via co-repressor recruitment. SERCAs covalently bind to an ER cysteine residue (C530), inhibiting the receptor activation and gene transcription. Selective ERβ agonists may be used to upregulate anti-proliferative signals in prostate cancer cells.

Regarding the endocrine treatment of PCa, other drugs were developed or are under study, such as anti-androgens [201,202], anti-AR [203,204,205], AR modulators [206,207] and degraders [208,209], glucocorticoid [210] and progesterone [211] receptor modulators, gonadotropin-releasing hormone agonists/antagonists [212], and steroid 17α-monooxygenase enzyme inhibitors [213,214]. This section will describe nuclear ER-directed molecules used for clinical treatment of PCa (Figure 4).

The use of tamoxifen for the treatment of PCa has been suggested since a long time ago [215,216]. A few preclinical studies showed that tamoxifen inhibits the growth of PCa cells and patients may benefit from the use of this drug in combination with immunotherapy, TGFβ, or Wingless and Int-1 (Wnt) antagonists [217]. Moreover, high-dose tamoxifen therapy was well tolerated in a heavily pretreated patient cohort with castratation-resistant PCa, where the drug demonstrated an inhibitory effect on cancer cell proliferation via suppression of phosphatidylinositol-4-phosphate 5-kinase-α/AKT and matrix metalloproteinase (MMP)−9/VEGF signaling pathways [218]. Optimistic results also arose from a phase II clinical trial testing the effect of another SERM, raloxifene, in combination with the anti-androgen bicalutamide [219]. Raloxifene has a similar half-maximal inhibitory concentration compared to tamoxifen (IC_50_: 2.9–5.7 nM and 3.0 nM, respectively [69]), hinting that the former could be suitable for clinical purposes. Another SERM, toremifene, led to a reduced fracture risk in PCa patients co-treated with denosumab, extending the list of compounds of this class potentially effective in PCa therapy, although it has a relatively high IC_50_ (1.0 µM) [69]. These data suggest that the clinical use of SERMs may be optimized for personalized PCa therapy, although complete regression does not occur when they are used alone. Despite these apparently positive results, clinical considerations should be carried out cautiously, since most of our knowledge about the use of SERM against PCa is based on in vitro experiments. Similar conclusions may be extended to the clinical use of fulvestrant as a treatment for PCa, taking into consideration that, in addition to ERα, inhibition of ERβ and agonistic activity at GPER were demonstrated as well [69,220,221]. This drug displays higher inhibitory activity on ERα than ERβ, as demonstrated with a lower IC_50_, which is 0.47 nM for ERα and 3.8 nM for ERβ [69]. Moreover, an in vitro study performed using LNCaP PCa cells suggested that fulvestrant could inhibit AR transcripts, decreasing the response to androgens [193]. Based on these premises, the drug was tested in clinical trials in men affected by androgen-independent PCa, with contrasting results, animating the debate about its real benefits [195,195,217,222,223]. Interestingly, encouraging data came from the combinatorial use of fulvestrant and immunotherapy, although in the context of breast cancer [224,225,226,227]. However, data from the treatment of PCa are still missing. An intriguing molecular mechanism of resistance to fulvestrant may be explained with the agonistic action on GPER [228]. This receptor is overexpressed in a series of tumors, including PCa, modulating proliferative and anti-apoptotic signals [229]. The use of fulvestrant could be linked to disregulation of GPER expression and signaling, providing the basis for tumor resistance.

PROTACs are synthetic heterobifunctional molecules forming complexes between target proteins and an E3 ubiquitin ligase, leading to ubiquitination and degradation of the target via the proteasome [230]. Among the various types of PROTACs available [231], compounds against ERα were recently described as a strategy possibly effective to degrade both wild-type and mutant receptor isoforms in breast cancer [232]. These molecules displayed ERα ubiquitination and degradation within the 0.1–10 µM range, as well as a “hook effect” at higher concentrations [233]. Interesting results were provided with in vitro studies, mainly from breast cancer cell lines, where PROTACs showed anti-proliferative effects and overall low toxicity [234]. These data encourage the use of PROTACs against PCa, given the consistent percentage of castration-resistant cases and positivity to ERα [235]. Another strategy for wild-type and mutant ERα targeting is provided by SERCAs [236,237]. These molecules exert antagonistic activity by binding covalently to an ERα-specific cysteine residue (C530) absent in other steroid hormone receptors. In preclinical studies, the SERCA H3B-6545 had consistent activity against endocrine-therapy-resistant tumors expressing wild-type or mutant *ESR1* transcripts [237], stimulating its testing into an ongoing clinical trial on breast cancer patients (NCT03250676). Moreover, in ERα-positive breast cell lines, SERCAs demonstrated an increased anti-proliferative effect via synergistic activity with cyclin-dependent kinases 4 and 6 (CDK4/6) or the mechanistic target of rapamycin (mTOR) inhibitors [236]. SERCAs are molecules developed recently. However, most of the endocrine-resistant tumors display ERα-dependent growth, suggesting that this novel class of antagonists could be interesting tools to be optimized for PCa clinical treatment [238]. Finally, a class of blockers of both ligand-independent transcriptional activation function 1 and 2 (AF1 and AF2) domains of nuclear receptors are CERANs. Studies on molecules with AF1 and AF2 inhibitory activity have been performed since several decades ago [239]. More recently, the CERAN OP-1250 has been available for oral administration. OP-1250 was tested in *ESR1*-positive preclinical models, where it induced complete ER degradation and inactivation, blocking gene transcription and cell growth, and demonstrated the capability to shrink brain metastases (reviewed in [240]). OP-1250 is under testing in a clinical trial for advanced and/or metastatic hormone-receptor-positive, HER2-negative breast cancer patients (NCT04505826). Finally, dual-mechanism ER inhibitors (DMERIs) were recently developed [241]. These molecules feature two distinct ER-targeting moieties, inducing noncanonical perturbations of the receptor structure. As a result, DMERIs alter the ER ligand-binding domain and stabilize multiple, dimeric antagonist substrates, effectively antagonizing the proliferation of ER-positive breast cancer cells. This tool provides a new, exciting strategy to potentially counteract breast cancer and PCa in the future.

New frontiers for the treatment of PCa consist in the recent development of ERβ agonists [242,243,244,245]. Oppositely to ERα, this receptor exerts anti-proliferative effects in the prostate, suggesting that ERβ agonists may be useful tools to counteract PCa, avoiding side effects of SERM [246]. The recently developed compound 8β-VE_2_ demonstrated efficacy in prolonging the life of breast cancer patients, when used in combination with tamoxifen [247]. The anti-proliferative role of this agonist was also tested in xenografted benign prostate hyperplasia and PCa tissues from men, where 8β-VE_2_ induced apoptosis of stromal and epithelial cells, leading to potential clinical opportunities [169]. Another ERβ agonist is erteberel, which has been tested in preclinical models of glioblastoma, revealing chemotactic potential exerted through mechanisms involving IL-1β, useful to promote innate immunity against cancer [248]. These results suggest that erteberel could be an interesting compound to be tested against PCa, but no data are still available in this regard. Finally, a recent study described the use of androgen synthesis blockers in combination with ERβ agonist soy isoflavones, in men affected by PCa [249]. The combination of these drugs was suggested as potentially beneficial to sustain ERβ-dependent signals, via a mechanism counteracting the EGFR migration to the nucleus, as a stimulus supporting cell proliferation. In this case, further studies are needed to understand how phytoestrogens targeting ERβ might prevent the development of tyrosine-kinase-driven cancer.

## 7. Conclusions

Growing evidence suggests that estrogen-related pathways are implicated not only in the development of breast cancer but also in PCa. Therefore, in addition to androgen-signaling pathways, transcriptional and non-transcriptional estrogen signaling may represent an opportunity for the treatment of advanced PCa. New compounds targeting specifically ERs led to advancements in the efficacy of therapies, although there is still a long way to go and resistance in cancer therapeutics is a problem to be faced in the next few years. Immunotherapies seem to lead to intriguing perspectives, although most PCa patients fail to respond to the clinical treatment. These approaches could find benefits from the combinatorial use of molecules targeting ERs, which may improve the efficacy of immunotherapies. Moreover, a deep characterization of the expression and role of specific splice variants in PCa subtypes may represent a new opportunity for the stratification of PCa patients and the development of splice-switching oligonucleotide (SSO) therapies.

## Figures and Tables

**Table 1:** The expression of ER isoforms and their roles in PCa.

Isoform	KDa	Expression in Prostate	Function in Prostate Cancer	Refs.
*ESR1* gene: Estrogen Receptor-α				
ERα-66 (full-length, often referred to as ERα)	66–67	Highly expressed in tumor stroma; ↑ expression in epithelium in PCa; ↑ hg-PIN; ↑ high Gleason score (GS) tumors; ↑↓ (?) CRPC.	Tumor-promoting.	[100,101,104,105,106,116,117,118]
ERα-46	46	Expressed in normal and malignant prostate tissue.	Not described in PCa.	[128,129]
ERα-36	36	Expressed in normal and malignant prostate tissue.	Not described in PCa.	[102,130,131,132]
*ESR2* gene: Estrogen Receptor-β				
ERβ1 (full-length, often referred to as ERβ)	59–60	Mostly expressed in prostate epithelial cells; ↓ hg-PIN; ↓ localized tumors; ↓ CRPC; ↓ high Gleason score (GS) tumors.	Tumor-suppressive.	[74,101,137,143,144,145]
ERβ2	55–56	Predominantly in the cytoplasm of prostate epithelial cells; ↑ in PCa, especially in the nucleus; ↑ metastatic PCa.	Tumor-promoting; promotes stem cell properties and the development of chemoresistance.	[137,143,144,145,146]
ERβ3	56	Not expressed in normal and malignant prostate cells.	Not applicable.	[134,143]
ERβ4	54	Expressed in normal and malignant prostate cells.	Not described in PCa; heterodimerizes with ERβ1 and enhances its transcriptional activity (from yeast two-hybrid and promoter luciferase assays).	[135,147]
ERβ5	53	Expressed in basal epithelial cells in benign prostate glands; ↑ metastatic PCa.	Tumor-promoting; promotes stem cell properties and the development of chemoresistance.	[135,137,143,145,146]

↑ increased expression; ↓ decreased expression; (?) contrasting results.
